# Reliability of Real-Time Kinematic (RTK) Positioning for Low-Cost Drones’ Navigation across Global Navigation Satellite System (GNSS) Critical Environments

**DOI:** 10.3390/s24186096

**Published:** 2024-09-20

**Authors:** Luca Tavasci, Francesco Nex, Stefano Gandolfi

**Affiliations:** 1 Department of Civil, Chemical, Environmental and Materials Engineering (DICAM), Alma Mater Studiorum, University of Bologna, Viale Risorgimento 2, 40136 Bologna, Italy; luca.tavasci@unibo.it; 2 Faculty of Geo-Information Science and Earth Observation (ITC), University of Twente, 7500 AE Enschede, The Netherlands; f.nex@utwente.nl

**Keywords:** drone navigation, autonomous infrastructure inspection, GNSS-denied environment, RTK, low-cost GNSS board

## Abstract

UAVs are nowadays used for several surveying activities, some of which imply flying close to tall walls, in and out of tunnels, under bridges, and so forth. In these applications, RTK GNSS positioning delivers results with very variable quality. It allows for centimetric-level kinematic navigation in real time in ideal conditions, but limitations in sky visibility or strong multipath effects negatively impact the positioning quality. This paper aims at assessing the RTK positioning limitations for lightweight and low-cost drones carrying cheap GNSS modules when used to fly in some meaningful critical operational conditions. Three demanding scenarios have been set up simulating the trajectories of drones in tasks such as infrastructure (i.e., building or bridges) inspection. Different outage durations, flight dynamics, and obstacle sizes have been considered in this work to have a complete overview of the positioning quality. The performed tests have allowed us to define practical recommendations to safely fly drones in potentially critical environments just by considering common software and standard GNSS parameters.

## 1. Introduction

The great majority of commercial and custom-made drones are nowadays installing one or more GNSS receivers on board to determine the position of the platform and safely navigate the outdoor environment according to pre-planned flight plans. Especially for small drones, it is fundamental to use compact and lightweight onboard GNSS (Global Navigation Satellite System) modules. Therefore, geodetic-class receivers are not suitable because of their weight and antenna dimensions, in addition to their cost. In this regard, several manufacturers have brought to the market an increasing number of lightweight and portable receivers in the last decade. Most of these instruments have increasingly high specifications, which evolved from stand-alone code-signal receivers to single- and double-frequency (and multi-constellation) RTK (Real-Time Kinematic) sensors.

RTK positioning [1] implies the use of two receivers, with the first set in a known position and acting as a master and the second receiver used as a rover to perform the survey. The rover should receive in real time both satellite signals and RTK corrections from the master. These corrections allow differencing the observable phase in real time, thus estimating the baselines (3D vectors) linking the two receivers and avoiding most of the impact of space-correlated error sources typically affecting GNSS signals [2]. Several low-cost GNSS modules have been delivered on the market in the last decade, mainly based on Ublox boards [3,4,5,6]. A typical limitation of these low-cost GNSS is their capability to acquire only the L1 carrier signal, while all GNSS satellites send carrier signals modulated over at least two different frequencies. Besides the higher number of observables that can be exploited, the use of different signal frequencies enables more efficient strategies to solve cycle slips, fix phase ambiguities, and deal with the ionospheric delay using proper linear combinations of observables [7,8,9]. Therefore, compared to single-frequency receivers, the double-frequency ones have a major advantage in those applications where the rover operates in critical conditions or several kilometers from the master station. The availability of a second signal and the related observables is also an advantage in terms of the redundancy of the system to be solved to retrieve the rover’s position.

The use of drones has surged in the last decades in several applications. Besides the classical surveying applications in open environments, drones have been increasingly used for the monitoring of infrastructure such as buildings or bridges. Despite the use of high-resolution cameras, these operations are normally executed very close to the construction for inspection, thus facing problems of multi-path and a reduced number of visible satellites. These monitoring activities require, therefore, both outdoor and indoor localization set-ups. The transition between these two environments is the most critical step, where we move from mainly satellite positioning to the integration of other sensors (e.g., inertial, visual, and range measurements) to safely navigate the drone.

In addition, these applications require precise real-time tracking of the drone, meaning a few centimeters of accuracy, where the full functionality of RTK positioning is mandatory. The failures of the positioning system can cause large errors, often not perceived by the pilot from the feedback received on the controller, which is a danger for the drone integrity and for the people in the surroundings. Besides the need to avoid collisions, precise localization is fundamental in several applications such as the close-range inspection of infrastructure, where cracks need to be precisely located.

Since April 2018, the Ublox company (Thalwil, Switzerland) has produced and sold F9P-class receivers. These are double-frequency and multi-constellation GNSS receivers that enhance the RTK performances allowed by the previous generation of low-cost devices [10,11,12,13,14]. In that regard, F9P instruments represent the state-of-the-art device to track a UAV in critical situations, and they are also fully portable on small drones and relatively cheap and affordable in most deployable solutions.

This paper aims to provide useful insights on the performance of these instruments in critical operational conditions, simulating situations where the number of satellites is strongly limited by the occlusions (or in some cases totally absent). In particular, goals of this paper are (i) to determine if the wrong positioning of the drone is recognizable in real time by any manual or automated controller (i.e., the pilot or any autonomous navigation algorithm can immediately notice it); (ii) to assess the time to recover (re-fixing the phase ambiguity) as soon as a sufficient number of GNSS signals are received, depending on different operational conditions; and (iii) to assess the capability of the system to prevent the “false-fixes” sometimes occurring after a signal outage, delivering wrong positions under apparently good working status.

To do so, an F9P RTK has been installed on a standard low-cost drone that many users own and might use for technical surveys. The output coordinates provided by the system through the well-known PX4-autopilot software, version 1.12 (https://px4.io/), set with standard parameters, have been analyzed to assess the GNSS module’s performances under various operating conditions. This paper does not focus on the best achievable RTK precision, which is reasonable at the centimeter level, but instead, it aims to cast light on the potential criticalities in kinematic positioning during a drone’s flight under critical conditions, close to the surveyed object, and in almost or totally GNSS-denied environments.

The issue of assessing or improving the performance of an RTK-piloted drone under GNSS-denied or critical environments has already been assessed by several authors. For instance, in [15], several sensors were implemented on a UAV to overcome denied GNSS limitations, such as IMU, pressure sensor, magnetometer, and pan-tilt-zoom camera. Also, in [16,17,18] visual tracking approaches were exploited to locate a drone without relying on GNSS. Drone navigation in challenging environments was tested in [19] using a heavy drone carrying a high-end inertial system. In [20], this issue was addressed by proposing a method based on the analysis of the DOP (Dilution of Precision) parameter to highlight potentially dangerous positioning performance of the GNSS, but only simulated data were used. In [21], an a priori 3D model and a point cloud of the environment were used to constrain the drone flight in conditions critical to the GNSS. Also, in [22], the navigation in a demanding urban environment was tested but using an expensive geodetic-class GNSS and coupling its data with inertial ones. In contrast, in this paper, we focus on lightweight UAVs that cannot be equipped with heavy GNSS antennas or high-end IMUs, looking at applications in unknown scenarios where such drones could be used for a first inspection. Moreover, we used real data and a real obstacle for satellite signals to test the RTK performance, exploiting experimental redundancy to spot anomalous results.

The paper is organized as follows: the developed methodology to test the instruments is described in Section 2. In Section 3, the performed tests and their results are described in detail, while a discussion of the achievements is reported in Section 4. Final remarks and conclusions, as well as possible future improvements to this study, are described in Section 5.

## 2. Materials and Methods

**Figure 1:**
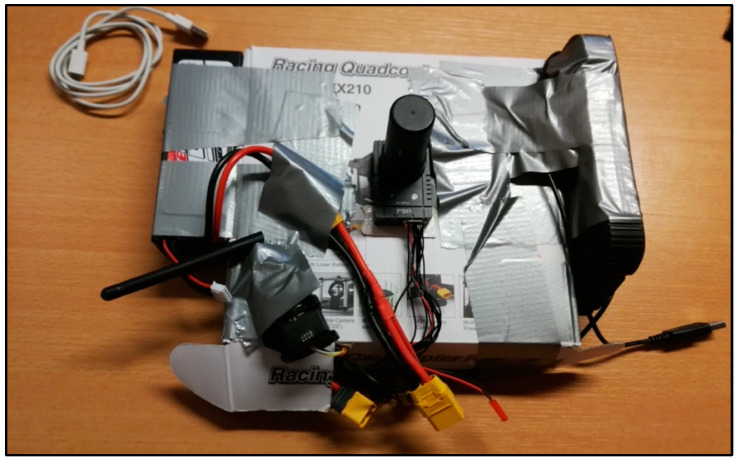
The Holybro Helical antenna mounted on the rover receiver based on the Ublox-F9P board, assembled with the power supply and radio transmission system all linked to the PX4 board inside the box.

The aim of the paper is to test the GNSS navigation performance in extreme conditions, as already mentioned. It was therefore decided to test the navigation system in a “controlled” setting, avoiding the flight of the drone. This setting had two advantages: (i) to make the tests more easily replicable and (ii) to prevent possible crashes of the drone platform. The GNSS was disassembled, taking apart the drone’s PX4 board, the RTK module, and the power supply (Figure 1), and then installed on a cart to better control the location and displacements. The cart was following predefined and reproducible paths, moving the receiver close to tall walls and under building tunnels (see the next section). This allowed us to repeat the same trajectories with an accuracy of a few (2 to 5) centimeters in the plane directions and less than 1 cm in the vertical one. This setting was considered sufficient to detect significant deviations (more than 10 cm) of the receiver localization during the tests. As mentioned, the GNSS board used in the test is the Ublox-F9P, built up and configured by the Holybro company (Hong Kong, China) in its “H-RTK F9P” kit (https://holybro.com/products/h-rtk-f9p-gnss-series accessed on 11 September 2024). In particular, the Helical antenna was chosen to be coupled to the rover receiver. In each test, the antenna position relative to the cart remained constant during data acquisitions. Possible rotations occurring on a real drone when accelerating and maneuvering were considered negligible since the Helical antenna used has no shield isolating signals coming from elevations lower than the cutoff angle. Moreover, for applications close to obstacles, a drone is likely flying at a slow speed with limited attitude changes.

In the performed tests, we decided not to generate signal interruptions by acting in post-processing on the raw data [23,24], but instead to simulate realistic working conditions to account for possible effects linked to multipath and changing sky visibility conditions. The data collection was carried out using the QGround Control software, version 4.2.0 [25], one of the well-known and most used packages for drone piloting, by recording the log files containing navigation parameters as they came from the RTK module. The master receiver was placed close to the test area (below 100 m distance) to avoid possible communication problems due to the radio connection. Moreover, the use of a master station as close as possible to the rover should ensure the most accurate estimation of the space-correlated GNSS biases. This makes the RTK operating conditions the best possible and leaves all possible sources of error to the inspected obstacles. Nevertheless, it is possible to navigate drones using Network RTK, which should provide corrections with similar quality and whose impact should be negligible compared to obstructions to the sky visibility and the multipath. Master’s coordinates were set up using the QGround Control standard procedure to initialize the navigation system, namely the “Survey-in” mode, with 3 min of observation time. This procedure leads the rover’s coordinates to be aligned to the WGS84 at the meter level of accuracy. Note that biases in the alignment to the reference frame due to the initialization process would equally affect all the coordinates of the same survey. Therefore, the intrinsic precision of the RTK positioning is preserved, whereas the surveys performed in different sessions cannot be compared at the centimeter level due to the re-initialization of the system. To have a survey aligned to the reference frame at the centimeter level, an official benchmark with known coordinates should be available in the area. Alternatively, an ad hoc benchmark should be surveyed using geodetic class GNSS receivers. Each of the tests described below was performed on the same day, in the same session, while keeping the master receiver in the same position, so that no issues due to the initialization process would bias the results. All tests were performed by repeating several runs (namely 24, 31, and 26) to guarantee a sufficient redundancy to calculate some statistics. Moreover, the choice of repeating the same test several times allowed evidencing unusual behaviors of the navigation system, which would not have been spotted by performing simpler testing, or, on the other hand, which would have dramatically affected the computed statistics. The full potentiality of the Ublox-F9P receiver was used, enabling data acquisition from the four available GNSS constellations, namely GPS, GLONASS, Galileo, and BeiDou. In the analysis of the results, there will be frequent reference to the working conditions of the RTK as “fix”, “float”, or “single”. It is worth noting that with the term “fixed solution”, we indicate the condition in which the receiver fixes as natural numbers the “initial phase ambiguities” of the GNSS carrier signals [26], thus allowing the system to work at its best precision. Without further details, the phase ambiguity is the number of whole cycles in the GNSS carrier signal that occurred between its departure and its reception by the receiver at the instant the satellite starts being tracked. This number remains constant over time until the satellite signal is lost, even if re-gained later, and constitutes one of the unknowns in the positioning problem. The condition of “float solution” implies the ambiguities to be defined only as real numbers, which usually leads to meter-level precisions, whereas what we will refer to as a “single solution” is technically a solution obtained without using phase observations. This latter case can be due to the impossibility of the rover receiving the master station’s corrections or lacking satellite signals. Finally, “multipath” [27] is the phenomenon that wrongly lengthens the measurement of the satellite–receiver distance because of signal reflection by metal/regular surfaces close to the GNSS antenna.

## 3. Testing and Results

Three testing activities were performed in different conditions, with the main purposes being to (i) find out whether the Ublox F9P RTK module is keeping the ambiguity fixing in the proximity of obstacles and occlusions that limit the sky visibility; (ii) assess the time to re-fix the solution after signal interruptions occurred, considering different operational conditions; and (iii) understand in which flying conditions false fixing or undetectable positioning gross errors occur more frequently. All these tests simulate common operational conditions faced by a UAV during any infrastructure inspection: in (i), the platform approaches the infrastructure, progressively reducing the number of visible satellites until the number of satellites is insufficient to deliver an accurate position; in (ii), the UAV platform moves away from the infrastructure (or an indoor environment) until it obtains a sufficient number of satellites to re-fix the ambiguity; and in (iii), the quality of the re-fixed ambiguity is assessed according to the motion of the UAV in front of the infrastructure.

**Figure 2:**
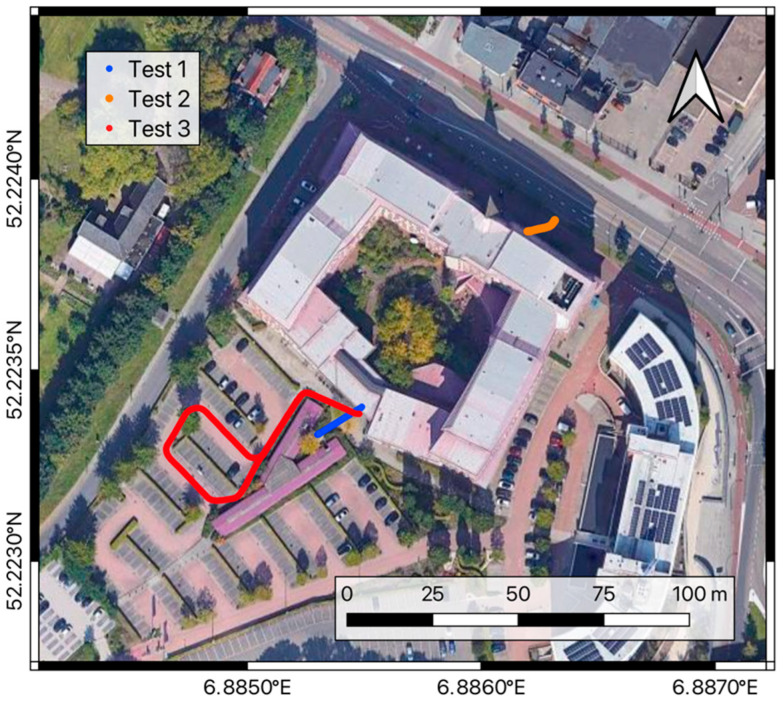
The ITC building tests site. The picture shows the tracks used for test-1, test-2, and test-3 in blue, orange, and red, respectively.

The testing activities were performed outside the old ITC Faculty building in Enschede, which offered tall walls causing multipath and obstructing the sky visibility (oriented both to North and South), and a short tunnel passing through the southwest façade, simulating an indoor (GNSS-denied) environment. Figure 2 shows details about the location of the three tests. For each of them, the three abovementioned aspects (i.e., keeping the ambiguity fixing, the time to re-fix, and the presence of false fixes) were inspected under different operational conditions.

In particular, test-1 and test-2 simulated the hovering conditions of a drone moving in and out of a tunnel and operating close to a tall wall of the building. In contrast, test-3 aimed to inspect the positioning performances of the drone flying under the tunnel and moving out of it, passing from partial sky visibility conditions and possible multipath to clear sky conditions.

### 3.1. Test-1: Hovering by a Tunnel Entrance

Test-1 was performed by running the cart carrying the navigation system back and forth from a position where the sky visibility was good enough to fix the phase ambiguities to a tunnel denying the satellite signals (blue dots in Figure 2) simulating the inspection of the bridge piles, which are otherwise hard to access without using a drone.

**Figure 3:**
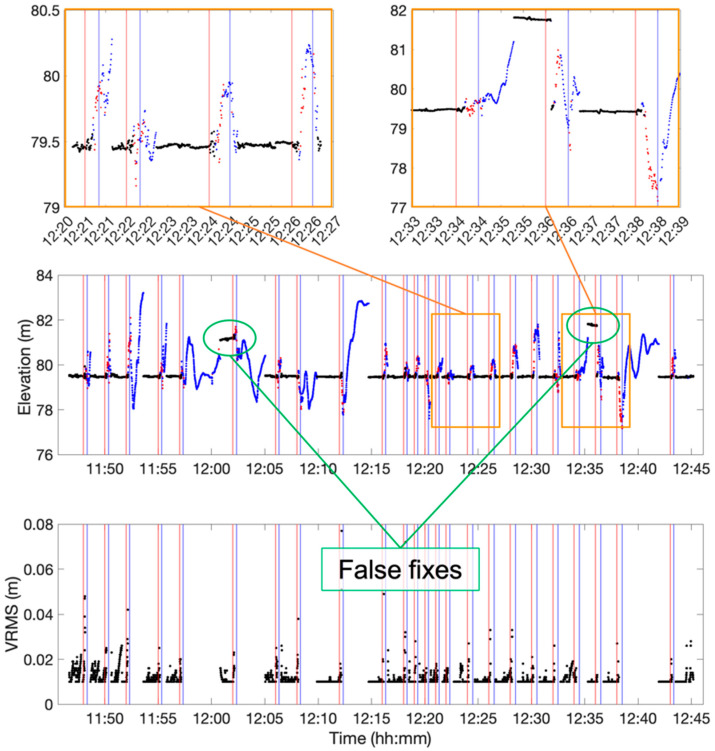
Time series related to test-1. The (**central**) chart shows the elevation (up direction). The black, blue, and red dots are used to represent fixed, floating, and single solutions, respectively. The red and blue lines represent the epochs when the cart started from the initial position and moved back from the tunnel, respectively. The two (**upper**) plots highlight typical cases from the time series. In particular, the left one shows the solution’s behavior under regular working conditions, whereas the left plot evidences a case of failure in the ambiguity fixing. The (**bottom**) chart shows the estimated vertical precision parameters related to the fix solutions only, while for float and single solutions, it goes by far out of scale.

The test began with the rover solutions in a fixed condition in a location where the sky visibility was good enough to see satellites in all directions, at least those with an elevation above 20 degrees. This starting position was close to a metal covered bike shelter that could have induced some multipath effect. Then, the cart was moved toward the building façade, which is about 7 m high in that position, and under the tunnel passing through it. Therefore, both a partial obstacle (the façade) and a total obstacle (the tunnel) to sky visibility occurred. After a time span ranging from 5 to 20 s under the tunnel, the cart was moved back to the initial position. This was repeated 24 times, always starting only once the receiver had re-gained the fixed condition. The cart was moved at a speed of about 1 m/sec. Figure 3 shows the time series of the elevation (up coordinates) recorded during the whole test, together with their estimated precision (bottom graph) in terms of RMS (1σ confidence level). Note that the estimated precision of the GNSS position provided by the QGround Control is split into the vertical and the horizontal directions, providing the VRMS and the HRMS, respectively. These precision estimates are also available in real time to the pilot and could in principle be used to decide how much trust to give to the positioning system. The red break lines represent the starting time of the cart from the initial position, whereas the blue ones indicate when the cart moved back from under the tunnel. The black dots represent the solutions obtained in fixed conditions, while blue dots refer to floating solutions and red ones indicate single solutions. The choice to show the vertical component in the time series is because this direction is the only one well constrained by the ground floor along the cart path, thus avoiding further variability of the coordinates other than coming from GNSS positioning errors.

**Figure 4:**
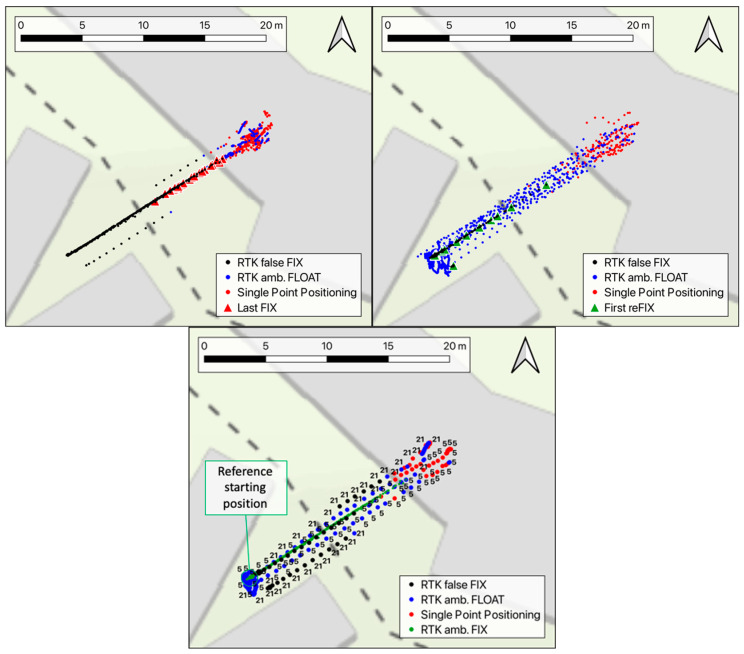
The chart reports the horizontal coordinates measured along the same path as in the test-1 runs. The black, blue, and red dots are used to represent fixed, floating, and single solutions, respectively. The (**top-left**) chart shows the runs from the starting position to the tunnel, while the trajectories of the way back to the initial position are reported in the (**top-right**) plot. Triangles show the positions of the last fixed solution approaching the tunnel or the first fixed solution on the way back to the initial position. The (**bottom**) chart focuses on the runs 5 and 21, which were affected by the false fixing of the phase ambiguities.

Figure 4 shows the horizontal trajectories of the rover, where the dots’ colors have the same meanings as in Figure 3. The top left plot represents the runs from the initial position to the tunnel, showing the overall good quality of the coordinates during the approach of the cart to the obstacle, even when the solutions are marked as floating (red dots). In contrast, in the top right chart, the runs back from the tunnel to the initial position are reported. In these cases, the significant difference between the trajectories for the floating solutions (blue dots) is evident. The full precision is resumed as soon as the fixing conditions are re-gained. This sometimes happens only after the return of the cart to the initial position. Note the partial obstruction given by the building façade, which makes the operational conditions sub-optimal along the path back to the starting position, which simulates a real scenario.

The bottom chart in Figure 4 focuses on a couple of runs, namely the 5th and the 21st, where cases of the false fixing of the initial phase ambiguities occurred. These can be also identified in Figure 3 (orange zoom-in in the right sub-figure), where the elevation error reaches about two meters. False fixes can be spotted since the distance between these positions to the other points marked as “fix” are far above the decimeter level, which is incompatible with the known repeatability of fixed ambiguity solutions (see Figure 15 and Figure 16 in [28,29,30]). Both cases occurred while the cart was moving away from the obstacle, and they lasted until it started the following run. The coordinates are also strongly biased in the horizontal components, especially for run 21.

**Figure 5:**
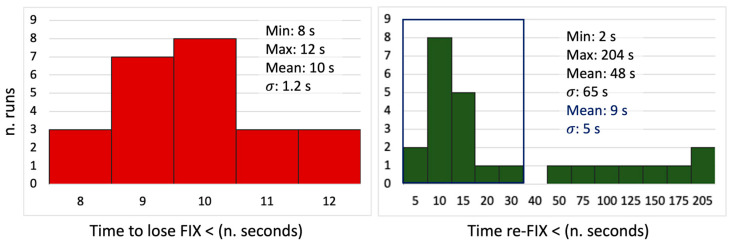
Statistics related to est-1. The (**left**) histogram reports the time to lose the ambiguity fixing condition starting from the initial position, while the (**right**) one shows the time required to re-gain the re-fix the solution after having left the obstacle.

Focusing on the time the system takes to lose and re-gain the fixing conditions, the histogram in Figure 5, on the left, reports the time (seconds) that passed between the start of the cart from its initial position and the instant the solutions switched from fixed to floating. On average, this time is 10 s (σ = 1.2). In Figure 4, top left, the positions where the solutions switch are marked using red triangles. This happens at variable distances from the entrance of the tunnel, mainly ranging from 0 to 5 m. The loss of fixing conditions before reaching the complete sky occlusion under the tunnel is due to the height of the building façade above the tunnel itself.

Figure 5 on the right reports the histogram of the times that the receiver needed to fix again the solution while going back to the initial position. This delay ranges between 2 and 204 s, and on eight occasions, it is longer than 25 s. No correlation between longer fixing delays and a poor number of satellites on sight was found. Nevertheless, cutting off these cases, the average time to re-fix is about 9 s, with a 5 s variability in terms of the RMS. In Figure 4, the positions where the fixing conditions were re-obtained by the system are depicted as green triangles. From this image, the fixing happened only when the cart was already back to the starting position in many cases. In these cases, the system needed several seconds to re-fix again, probably because of both the presence of the main building façade, which partially obstructs the sky and the multipath caused by the metal shelter around the point.

### 3.2. Test-2: Hovering Close to a Tall Wall

The second test differs from the first one for the obstacle used to obstruct the sky visibility. In this test, the almost-30-meter-high north-east façade of the ITC building (see orange dots in Figure 2) was used. Note that the impact of such an obstacle is increased at the test site’s latitude (52 degrees) due to the hole in GNSS constellations above the poles (see the orbital plane details in [31]), which leaves no satellites orbiting at mid–low elevations in the north direction, the one left free from the test obstacle. In this condition, the impact of the satellite geometry on the positioning accuracy is higher. This makes the test scenario one of the worst possible in the hovering of a drone close to a tall wall simulating the case in which a drone is used in the inspection of a rock quarry wall or a tall concrete dam. In this test, 31 runs were performed, leaving the receiver against the wall for 15 s in the first ten runs, 30 s in the runs 11–20, and 75 s in the last runs, except for the 29th. The approaching speed of the cart was in the order of 1 m/s.

**Figure 6:**
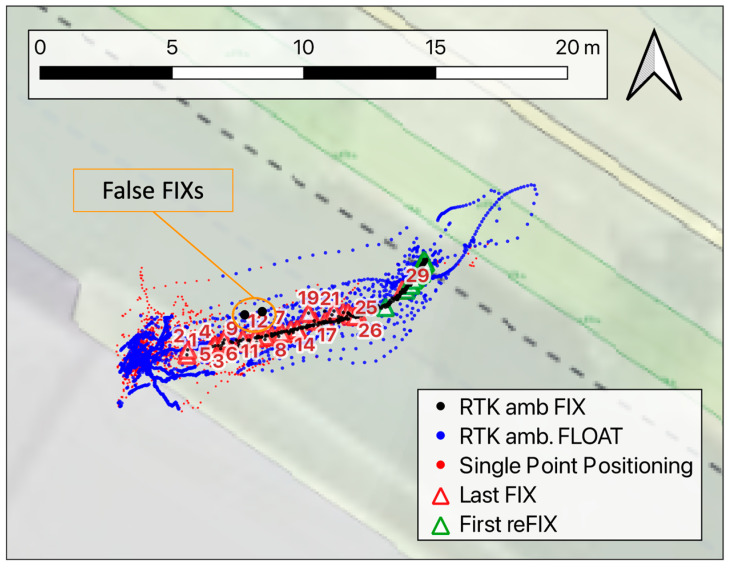
Map of the horizontal coordinates surveyed during the test. The black, blue, and red dots are used to represent fixed, floating, and single solutions, respectively. The triangles show the positions of the last fixed solution approaching the building façade or the first fixed solution on the way back to the initial position. The numbers close to the red triangles are related to the test runs.

Figure 6 reports the horizontal coordinates surveyed during the test. In these pictures, black, blue, and red are again used to represent the fixed, floating, and single solutions, respectively.

**Figure 7:**
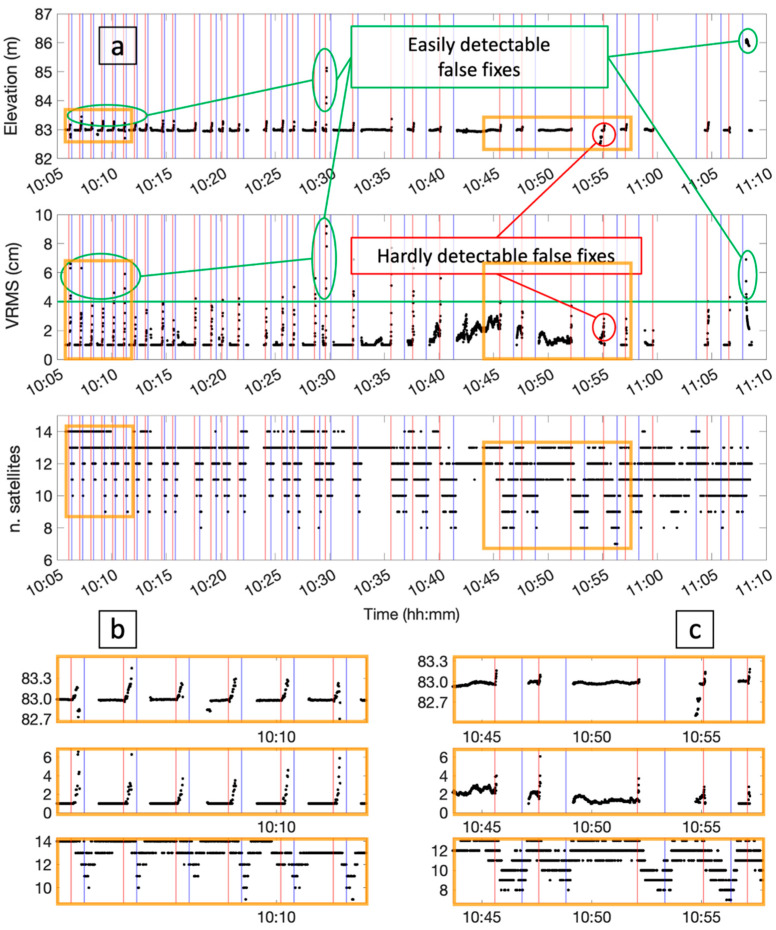
(**a**): The top chart in reports the time series of the up coordinates recorded in ambiguous fixed conditions. The estimated vertical precision is shown in the middle plot, whereas in the bottom, the time series is the number of GNSS satellites in sight of the receiver during the test. (**b**) is a zoom-in of the runs where the estimated error VRMS is well correlated to the real error in height, while (**c**) highlights the test results in a period with fewer satellites in sight and some trouble (see minute 10:54) for the VRMS in perceiving false fix solutions.

The red triangles represent the last fixed solution of each run, while the green ones denote the first ambiguity of fixed solutions on the way back of the cart. Also in this test, the fixing conditions were lost in different positions for the different runs, mainly within 5 m from the obstacle, whereas the re-fix occurred most of the time close to the initial position of the cart. More specifically, the fixing condition was lost 1–2 m far from the wall in the first runs, while later, this position moved back to 5–6 m (see the run numbers in Figure 6). This variability in this position may be due to the changing satellite constellation: by looking at the bottom time series plotted in Figure 7a, which reports the number of GNSS satellites in sight at each measuring epoch, this value varies between 7 and 14 and tends to decrease over time. This probably causes the higher impact of the obstacle on the ambiguity fixing in the late runs. It is worth noting that in test-2, the obstacle is much higher than in test-1, thus causing more of a multipath effect during the approach to it, while on the other hand, it does not constitute a complete occlusion to the sky visibility.

Figure 7a also shows the up component of the fixed solutions only (top) and the estimated vertical precision VRMS (middle time series). As for Figure 3, the vertical component is shown, as it is the best constrained along the cart path. Looking at the zoom-in in Figure 7b, it can be noticed that right before switching to the float condition, the accuracy of the coordinates strongly worsened. Nevertheless, the VRMS parameter appears to be quite representative of the actual positioning quality and thus might be used to highlight possibly unreliable fix solutions. Some false fixed solutions occurred during the test, as highlighted both in Figure 6 and in the top plot of Figure 7a. In such cases, the VRMS coherently increased at the measuring epochs of the outlier solutions. In contrast, the zoom-in in Figure 7c highlights the case of a false fix bringing an error of about 40 cm in height (around 10:55), which cannot be highlighted by looking at the related HRMS. The time span considered in Figure 7c is in general characterized by a lower number of satellites in sight; this also caused a slightly less stable estimation of the height position even for the good solutions, as properly evidenced by the VRMS.

**Figure 8:**
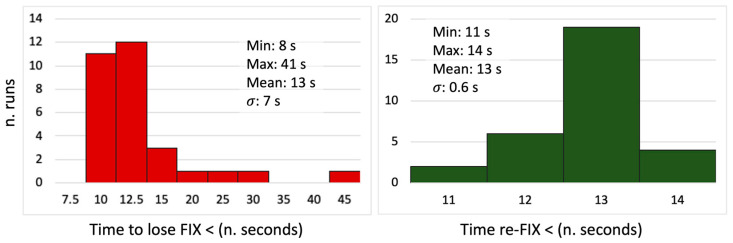
Statistics related to test-2. The (**left**) histogram reports the time to lose the ambiguity fixing condition starting from the initial position, while the (**right**) one shows the time required to re-gain the re-fixed the solution after having left the obstacle.

Figure 8 reports histograms and statistics of the time the system took to lose and re-gain the fixing condition. To re-fix the solution, the receiver took on average 13 s, with a rather small variability in the data, with a standard deviation of less than one second. These values were calculated considering the time from the instant the cart passed the average position where the GNSS fixing were lost in the one-way runs.

### 3.3. Test-3: Flight in Changing Conditions

**Figure 9:**
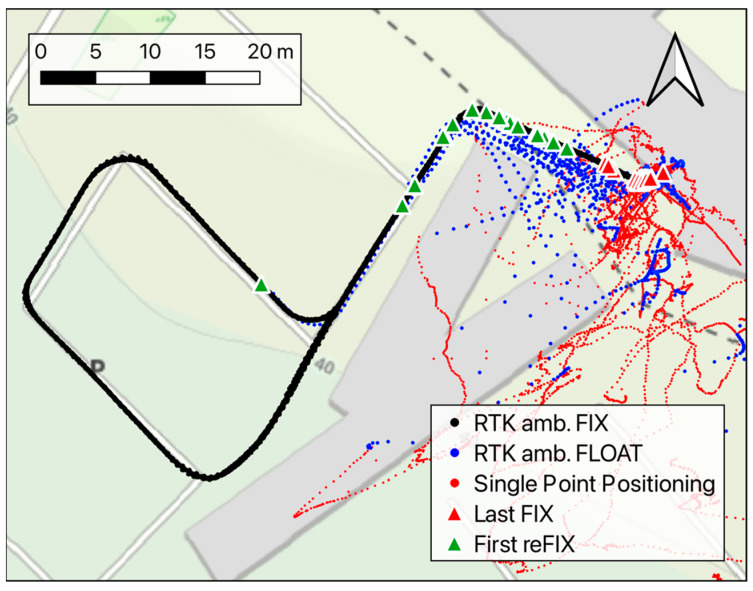
Horizontal coordinates recorded during test-3 in fixed (black dots), floating (blue dots), and single-point positioning (red dots) conditions. The red and green triangles highlight the last and first fixed solution, respectively, for each run.

The third test focuses on the behavior of the RTK positioning system under kinematic conditions, meaning a drone that keeps moving after passing through a GNSS-denied spot instead of hovering close to the obstacle. This condition may be representative of a drone surveying a road or a railway and passing under a wide bridge, a short tunnel, or through an urban canyon between buildings. Also, the case of the exit after an indoor flight can resemble this test. As shown in Figure 2 (red dots), the cart carrying the RTK rover receiver followed a circuit passing under the tunnel of the ITC building’s south entrance, then flanking the lowest part of the building wall for about 20 m, and finally moving in the parking lot where the sky visibility is almost optimal. For this test, the approximate moving speed of the cart is about 2 m/s. The track was covered 26 times, and Figure 9 shows the plane coordinates recorded during the test. As in the previous tests, the black, blue, and red dots represent fixed, floating, and single-point positioning solutions, respectively, whereas the triangles are used to show the positions where the ambiguity fixing was lost or re-gained. In this case, the receiver lost the fixing in positions a few meters from each other, near the entrance of the tunnel, while a rather bigger variability can be observed in the position where the receiver re-gained the fixing condition.

**Figure 10:**
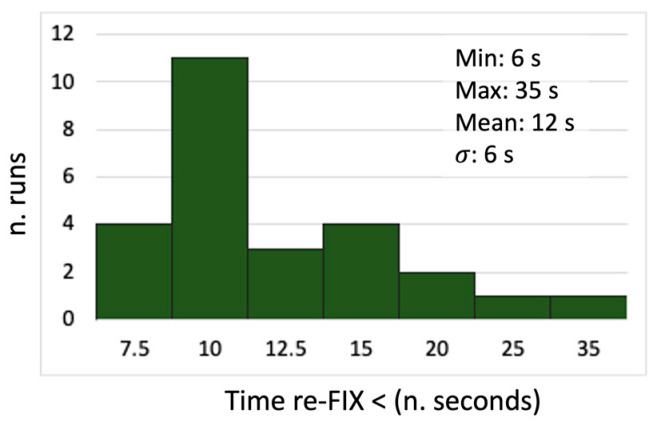
Statistics related to test-3 about the time required to re-gain the re-fixed solution after having left the tunnel.

In terms of time to re-fix the solutions after having left the tunnel, the main results are shown in Figure 10 and were calculated relative to the instant when the receiver passed on the average position in which the fixing condition was lost approaching the tunnel. These time spans ran from 6 to 35 s, where the latter value was quite an exception; excluding it, the average time was about 11 s with a variability of 4 s RMS.

Additional tests were performed by varying the time under the tunnel to assess the influence of the time interval without satellite signals. A first attempt was to vary the time spent in an urban canyon or under a bridge to verify its influence on the re-fixing time.

For this reason, the cart was left under the bridge for a time span of 10 and 30 s for the first and second tens of runs, respectively, while during the last six runs, this time was one minute. These intervals were selected considering a typical operational situation, where the drone has limited flight time, and inspections cannot last too long without dedicated sensors (such as LiDAR) and SLAM algorithms [32,33] for indoor navigation. However, these tests did not highlight any relevant changes in the receiver’s performance, depending on the time spent in signal-denied conditions, at least for the relatively limited time intervals considered.

No significant false fixes were detected in test-3, which may indicate that the receiver motion helped the system avoid keeping a wrongly estimated set of initial phase ambiguities to define the fix conditions. This is reasonable, as a set of integer ambiguity values, when correctly estimated, should remain coherent as the receiver position change, while motion emphasizes its incoherence, since both the initial-phase ambiguities and receiver coordinates are unknown for the problem estimated at once.

## 4. Discussion

We assume that the goal of the navigation system is to provide real-time positions that are accurate within the 10-centimeter level. The performed tests gave relevant information on the behavior of a state-of-the-art GNSS installed on a lightweight UAV. As is already known, only RTK GNSS modules allow such a positioning accuracy, while stand-alone (i.e., single-point) positioning solutions can only generate metric accuracies, if not worse.

RTK will enable the real-time differencing of the observable phase so that kinematic baselines between the master and the rover receiver can be calculated. In that regard, it is fundamental for the pilot to have the real-time information concerning the status of the ambiguity fixing, since only with the set of phase ambiguities fixed as natural numbers can the system provide the required accuracies, while in the floating conditions, the errors can reach the meter level or more.

In almost all the performed tests, a solution marked as an “ambiguity fix” provides reliable coordinates at a precision level of a few centimeters. In a few cases, tests evidenced the so called “false fix”, meaning the cases when the statistical algorithm used to choose the set of natural numbers for the initial phase ambiguities failed. In these cases, despite the “fix” mark on the solutions, the given coordinates may have had errors at the meter level, making this condition one of the most dangerous for a drone flight close to obstacles. False fixes occurred during test-1 and test-2, which simulate the hovering condition of a drone, while no evident failure on the ambiguity fixing was observed in test-3. This is because a set of wrong phase ambiguities became statistically incoherent when the receiver changed its position: the moving condition prevented the system from keeping a false fix. In contrast, hovering drones in the same position led the GNSS system to ignore these inconsistencies: in this case, only the change in the satellite positions can reveal a wrongly defined set of initial phase ambiguities, but this takes several minutes. In principle, to shorten this time, the drone should move sufficiently to cause variations of a whole wavelength cycle along the distance between the receiver and all satellites: in this case, a wrongly estimated set of ambiguities could reveal its incoherency. In practice, this could be reasonably obtained with a displacement in the vertical direction larger than 0.5 m or a 1-meter shift in planimetry.

According to the performed tests, there are no easy and reliable ways to recognize a false fix in real time. In most cases, like the ones highlighted in Figure 7, related to test-2, the real-time estimation of the positioning accuracy (which the software provides in terms of HRMS/VRMS) is a good proxy to determine false fixes. These parameters are correlated with the satellite geometry (DOP, number of satellites), in addition to multipath and other parameters, and they constitute an attempt to estimate the positioning quality. Thus, they can be exploited by setting a threshold to define the unsuitability of the RTK solution: from the performed tests, a 4 cm (see the green line in Figure 7 central plot) value could be a balanced choice to reject false positives without being too small to exclude reliable solutions, too. Reasonable values could range between 3 and 5 cm. A higher positioning quality should be guaranteed by a lower value, with the drawback of considering unreliable correct solutions with higher probability. In contrast, setting the threshold to five should move the balance to a less conservative choice, also accepting solutions more prone to significant errors.

As shown in Figure 3 and Figure 7, whenever the drone approaches the obstacle and there is a transition from fixed to floating conditions, the last coordinates surveyed before the switch are characterized by comparatively high errors (decimeters). These cases are due to the lack of GNSS signals or poor signal quality due to the multipath and sky obstruction. By looking at the bottom plots in Figure 3 and Figure 7 reporting the VRMS, it can be noticed that in these cases, such parameters evidence the deterioration of the coordinate’s quality. Therefore, using a threshold on these parameters would help the navigation system to become aware of the approaching critical conditions, too.

In terms of the time to recover the ambiguity-fixed condition after flying far enough from an obstacle, the tests showed that it is usually necessary to wait about 9–13 s. These time spans were calculated with respect to the instant the cart passed the average position where the GNSS fixing was lost approaching the obstacle and considering the drone moving back at a walking speed (about 1–2 m/s). The presence of tall walls (in test-2 and test-3) negatively impacted the time to re-fix the solution. In contrast, in test-1, the boundary conditions in terms of surfaces inducing a multipath caused the occurrence of some significantly longer fixing times. Table 1 summarizes three practical recommendations that drones users should consider when flying in conditions critical to the sky visibility.

Finally, it is worth noting that the RTK-positioning technique is a kind a three-dimensional relative positioning of the rover receiver with respect to the master one. Therefore, when the RTK works properly, the drone’s positions are coherent between themselves at a level of a few centimeters, but their overall accuracy also depends on the quality of the coordinates assigned to the master station. This means that, in the case of the automatic flying of the drone, following a pre-defined plan around the surveyed object, the coherence of the master’s coordinates to those used to survey the object itself is fundamental. This can be achieved by setting the master station over a benchmark surveyed with centimeter-level accuracy, potentially using a geodetic-class GNSS receiver or a whatever topographic technique linking its coordinates to the same reference system used to plan the flight.

## 5. Conclusions

This technical note aims to provide readers with insights about the performances of a GNSS/RTK module when used to locate with centimeter-level precision a drone flying in critical environments, with a reduced or no GNSS signal. In particular, we tested a module based on the Ublox F9P receiver, mounted on a cart so that it was possible to move it in critical positions where GNSS signals are denied. The coordinates and ancillary parameters recorded in the QGround Control log files were analyzed to better understand the behavior of the receiver. Three main tests were conceived to simulate practical scenarios of a drone flying close to, or under, human-made objects that partially or completely obstruct the sky, thus denying satellite signals.

The performed tests give drone users some practical and relevant insights into the quality of the GNSS positioning in these conditions. Only fixed solutions can deliver accurate values of the drone’s position. HRMS/VRMS parameters have been shown to be good proxies to determine wrong or low-quality positions and should be accurately considered in flying from outdoor to indoor conditions. Another important achievement of our tests is that the hovering flight of the drone does not help the re-fixing of the phase ambiguities, making the solution more prone to wrong localizations.

The tests were performed using the master station within a hundred meters, as is the case in most real cases with drones. In this regard, the use of double-frequency receivers gives similar behaviors in terms of fixing time for larger distances, too. Nevertheless, depending on the hardware used, the radio link between the master and the rover might become the limiting factor to the RTK positioning quality. To provide drones users with more comprehensive results, different baseline setups, and a broader set of locations, simulated obstacles and GNSS antennas could be analyzed.

The experiments performed in this case study were limited to the assessment of the positioning quality, considering the few parameters normally available in QGround Control and other drone software solutions. This was carried out to keep the results close to the practical experience of experts assembling and delivering drone solutions. However, more precise reference solutions (for the cart trajectories), as well as a more thorough analysis of parameters such as DOP, constellation sky-plots, and multi-path influence, would have probably improved the presented analysis, despite not adding further practical insights to the results of this work.

## Figures and Tables

**Table 1:** Good practices to be implemented in a drone flight under potentially critical conditions for the GNSS navigation system.

Necessity	Possible Solution
Navigate the drone with a precise positioning (coherence of the coordinates within 10 cm).	Use only RTK solutions marked as “fix”, not considering those acquired under “float” or “single” conditions.
Avoid trusting “fix” solution possibly affected by significant errors (>10 cm, up to meters).	Set a threshold on the HRMS/VRMS parameters (3 to 5 cm), and reject exceeding solutions.
Avoid “false fix” potentially occurring after re-fixing the solution out of a GNSS-denied environment.	Keep the drone moving right after re-fixing the phase ambiguities (1 m or more in plan, or 0.5 m vertically) before approaching the obstacle again instead of slowly hovering close to it.

## Data Availability

Data are contained within the article.

## References

[1] Huang G., Du S., Wang D. (2023). GNSS techniques for real-time monitoring of landslides: A review. Satell. Navig..

[2] Karaim M., Elsheikh M., Noureldin A., Rustamov R.B. (2018). GNSS error sources. Multifunctional Operation and Application of GPS.

[3] Groves P.D., Adjrad M. (2019). Performance assessment of 3D-mapping-aided GNSS part 1: Algorithms, user equipment, and review. Navigation.

[4] Lu L., Ma L., Wu T., Chen X. (2019). Performance analysis of positioning solution using low-cost single-frequency u-blox receiver based on baseline length constraint. Sensors.

[5] Mongredien C., Doyen J.P., Strom M., Ammann D. Centimeter-level positioning for UAVs and other mass-market applications. Proceedings of the 29th International Technical Meeting of the Satellite Division of the Institute of Navigation (ION GNSS+ 2016).

[6] Poluzzi L., Tavasci L., Corsini F., Barbarella M., Gandolfi S. (2020). Low-cost GNSS sensors for monitoring applications. Appl. Geomat..

[7] Robustelli U., Paziewski J., Pugliano G. (2021). Observation quality assessment and performance of GNSS standalone positioning with code pseudoranges of dual-frequency Android smartphones. Sensors.

[8] Teunissen P.J., Jonkman N.F., Tiberius C.C.J.M. (1998). Weighting GPS dual frequency observations: Bearing the cross of cross-correlation. GPS Solut..

[9] Cai C., Liu Z., Xia P., Dai W. (2013). Cycle slip detection and repair for undifferenced GPS observations under high ionospheric activity. GPS Solut..

[10] Mahato S., Santra A., Dan S., Banerjee P., Kundu S., Bose A. (2023). Point positioning capability of compact, low-cost GNSS modules: A case study. IETE J. Res..

[11] Pavlovčič-Prešeren P., Dimc F., Bažec M. (2021). A comparative analysis of the response of GNSS receivers under vertical and horizontal L1/E1 chirp jamming. Sensors.

[12] Zahradník D., Vyskočil Z., Hodík Š. (2022). Ublox F9P for geodetic measurement. Stavební Obz.-Civ. Eng. J..

[13] Poluzzi L., Gandolfi S. (2021). Performance of Dual-Frequencies Low-Cost GNSS Sensors for Real Time Monitoring. Italian Conference on Geomatics and Geospatial Technologies.

[14] Sanna G., Pisanu T., Garau S. (2022). Behavior of low-cost receivers in base-rover configuration with geodetic-grade antennas. Sensors.

[15] Chen C., Tian Y., Lin L., Chen S., Li H., Wang Y., Su K. (2020). Obtaining World Coordinate Information of UAV in GNSS Denied Environments. Sensors.

[16] Chowdhary G., Johnson E.N., Magree D., Wu A., Shein A. (2013). Gps-denied indoor and outdoor monocular vision aided navigation and control of unmanned aircraft. J. Field Robot..

[17] Tang Y., Hu Y., Cui J., Liao F., Lao M., Lin F., Teo R.S. (2018). Vision-aided multi-uav autonomous flocking in gps-denied environment. IEEE Trans. Ind. Electron..

[18] Kim Y., Jung W., Bang H. (2014). Visual target tracking and relative navigation for unmanned aerial vehicles in a gps-denied environment. Navigation.

[19] Elamin A., Abdelaziz N., El-Rabbany A. (2022). A GNSS/INS/LiDAR Integration Scheme for UAV-Based Navigation in GNSS-Challenging Environments. Sensors.

[20] Isik O.K., Hong J., Petrunin I., Tsourdos A. (2020). Integrity Analysis for GPS-Based Navigation of UAVs in Urban Environment. Robotics.

[21] Zimmermann F., Eling C., Klingbeil L., Kuhlmann H. (2017). Precise positioning of uavs–dealing with challenging rtk-gps measurement conditions during automated UAV flights. ISPRS Ann. Photogramm. Remote Sens. Spat. Inf. Sci..

[22] Li T., Zhang H., Gao Z., Chen Q., Niu X. (2018). High-Accuracy Positioning in Urban Environments Using Single-Frequency Multi-GNSS RTK/MEMS-IMU Integration. Remote Sens..

[23] Jouybari A., Bagherbandi M., Nilfouroushan F. (2023). Numerical Analysis of GNSS Signal Outage Effect on EOPs Solutions Using Tightly Coupled GNSS/IMU Integration: A Simulated Case Study in Sweden. Sensors.

[24] Havyarimana V., Xiao Z., Semong T., Bai J., Chen H., Jiao L. (2021). Achieving reliable intervehicle positioning based on redheffer weighted least squares model under multi-GNSS outages. IEEE Trans. Cybern..

[25] Dardoize T., Ciochetto N., Hong J.H., Shin H.S. (2019). Implementation of ground control system for autonomous multi-agents using qgroundcontrol. 2019 Workshop on Research, Education and Development of Unmanned Aerial Systems (RED UAS).

[26] Blewitt G. (1989). Carrier phase ambiguity resolution for the Global Positioning System applied to geodetic baselines up to 2000 km. J. Geophys. Res. Solid Earth.

[27] Xie P., Petovello M.G. (2014). Measuring GNSS multipath distributions in urban canyon environments. IEEE Trans. Instrum. Meas..

[28] Robustelli U., Cutugno M., Pugliano G. (2023). Low-Cost GNSS and PPP-RTK: Investigating the Capabilities of the u-blox ZED-F9P Module. Sensors.

[29] Janos D., Kuras P. (2021). Evaluation of Low-Cost GNSS Receiver under Demanding Conditions in RTK Network Mode. Sensors.

[30] Janos D., Kuras P., Ortyl Ł. (2022). Evaluation of low-cost RTK GNSS receiver in motion under demanding conditions. Measurement.

[31] Kaplan E.D., Hegarty C. (2017). Understanding GPS/GNSS: Principles and Applications.

[32] Kukko A., Kaijaluoto R., Kaartinen H., Lehtola V.V., Jaakkola A., Hyyppä J. (2017). Graph SLAM correction for single scanner MLS forest data under boreal forest canopy. ISPRS J. Photogramm. Remote Sens..

[33] Macario Barros A., Michel M., Moline Y., Corre G., Carrel F. (2022). A Comprehensive Survey of Visual SLAM Algorithms. Robotics.

